# Synovial Macrophages in Rheumatoid Arthritis: The Past, Present, and Future

**DOI:** 10.1155/2020/1583647

**Published:** 2020-04-13

**Authors:** Jiajie Tu, Xinming Wang, Xun Gong, Wenming Hong, Dafei Han, Yilong Fang, Yawei Guo, Wei Wei

**Affiliations:** Institute of Clinical Pharmacology, Anhui Medical University, Key Laboratory of Anti-Inflammatory and Immune Medicine, Ministry of Education, Anhui Collaborative Innovation Center of Anti-Inflammatory and Immune Medicine, Hefei, China

## Abstract

The ontogeny of macrophages in most organs has already been established. Owing to the limited number and inaccessibility of synovial macrophages (SMs), the origin of SMs has not been fully elucidated. Previous studies suggested that SMs have two major origins, namely, tissue-resident and monocyte-derived SMs. However, no systematic analysis to identify SM ontology in either physiological or pathological conditions has been available to date. In this review, we summarize relevant studies on the two main origins of SMs in rheumatoid arthritis (RA) and forecast the future research directions for this field. Furthermore, we discuss the current state of RA therapy that is based on targeting different SM subsets.

## 1. Previous Perspective

In 1967, Takasugi and Hollingsworth found a group of large, phagocytic cells (macrophage-like cells) in fluids from rheumatoid arthritis (RA) patients [1]. With the development and advancement of synovial needle biopsy and synovectomy, the removed joint synovium has become available for systematical experiments [2]. Since then, several studies have investigated the role of synovial macrophages (SMs) in RA. Macrophages produce various cytokines and chemokines, and they are also involved in cartilage and bone destruction, which can critically contribute to the pathogenesis of RA [3].

The healthy joint synovium is a thin piece of tissue that only contains a few layers of cells. However, the hyperplastic synovium from an RA patient has two clearly thick layers, known as the lining and sublining layers, both of which are abundant in SMs that are positioned throughout the two layers at the cartilage-pannus junction and mediate articular destruction. SMs are now also considered as a reliable biomarker for evaluating RA severity and response to RA therapy [4]. Using the replaced joint synovium from RA patients, Mulherin et al. [5] showed that the number of SMs positively correlate with articular destruction. The RA synovium contains numerous HLA-DR^+^ SMs in both the synovial lining and sublining layers, suggesting that SMs are activated in RA synovium through antigen presentation [6]. In addition, mature macrophages that also function as APCs may be needed to maintain a normal immune response in RA patients following B cell depletion by rituximab treatment [7]. These preliminary studies emphasized the essential role of SMs in RA (Table 1).

Basically, two main sources of macrophages were identified, namely, tissue-resident macrophages and circulating-derived macrophages. Tissue-resident macrophages exhibit a distinct phenotype that is linked to the corresponding functions of corresponding tissue, such as Clec4f^+^ Kupffer cells (KCs) (liver-resident macrophages) in the liver [8]. In addition, macrophages were originally proposed to be derived from circulating monocytes. These Ms4a3^+^ circulating-derived macrophages also play an important role in inflammation state [9]. Emerging evidence has shown that the function of SMs in RA is highly complicated due to the different subsets of SMs in the RA synovium. In general, CD14 and myeloid-related proteins (MRP) 8 and 14 are considered as markers of a circulating monocyte-macrophage lineage in RA synovium. For synovial-resident SMs, no well-acknowledged markers have been detected to date. CD68 and CD163 are more highly expressed in resident SMs than infiltrating circulating SMs in RA synovium. However, similar previous studies have shown contradictory results. De Rycke et al. [10] demonstrated that infiltrating SMs from circulation that express MRP8 and MRP14, as well as resident SMs (CD163^+^), are abundant in the inflamed synovium. The numbers of CD14^+^/CD68^+^ SMs decrease in the synovial lining layer, whereas they remain stable in the sublining layer, suggesting that resident SMs mainly localize in the lining layer. Ambarus et al. [11] further showed that intimal lining layer SMs but not synovial sublining SMs display an M2-like phenotype in chronic synovitis, indicating that resident SMs in the lining layer show anti-inflammatory features. However, infiltrating circulating SMs are also upregulated in the lining layer [10]. Therefore, the lining layer not only contains resident SMs but also contains high levels of circulating SMs. Interestingly, *in vivo* treatment with anti-TNF-*α* has been found to exhibit a rapid and pronounced effect on the infiltration of MRP^+^ circulating SMs into tissues, while not affecting resident SMs [10]. The different response of resident and circulating SMs to TNF-*α* further highlights the different cellular functions and responses of these two subsets of SMs to RA drugs.

In addition, some other studies have suggested that the current markers for circulating and resident SMs are not adequately specific. Fonseca et al. [12] observed all CD163^+^ SMs as CD14^+^ cells in the synovium. Fewer cells were labeled with CD163 than with CD68 antibody in the synovial intima; however, all CD45^+^ intimal cells were CD163^+^. Based on this study, it seems that CD68 is a more reliable marker for resident SMs than CD163. In addition, CD4^+^IFN^+^ T lymphocytes in the RA synovium were chiefly localized within clusters containing CD68^+^CD163^−^ cells, suggesting that specific interactions may exist between IFN^+^ T cells and CD68^+^ SMs in the RA synovium. Greisen et al. found that soluble macrophage-derived CD163 is a marker of disease activity and progression in early RA [13], and soluble CD163-labeled SMs show different responses to synthetic and naturally occurring disease-modifying antirheumatic drugs (DMARDs) [14]. The number of CD14^+^CD3^−^CD19^−^CD56^−^ monocytes/macrophages was repressed in the synovial fluid mononuclear cells (SFMCs) of RA patients compared to those of gout patients. In this study, CD14^+^ cells showed a phenotype characteristic of circulating monocytes rather than tissue-resident SMs, characterized by high expression of CCR2, MRP8, and MRP14, but low expression of MERTK and 25F9. These cells had the capacity to produce proinflammatory cytokines. In addition, anti-inflammatory features, including CD163 expression and IL-10 production from CD14^+^ cells, were inhibited in RA patients more prominently compared to those with gout. CD14^+^ cells of the M2 macrophage phenotype also exhibited high phagocytic activity for monosodium urate crystals. Therefore, CD14^+^ monocytes/macrophages exhibited different subsets characterized by proinflammatory and anti-inflammatory characteristics [15]. Therefore, current SM markers may not be adequately specific to distinguish between the circulating and resident SMs. It is also difficult to further divide these two subsets of SMs into smaller groups with different functions, such as proinflammatory or anti-inflammatory SMs, using these markers alone.

Except macrophage markers in human, some other macrophage markers were used to identify the different subsets of SMs in mice. Ly6C is a murine marker for circulating monocytes. Ly6C^+^ monocyte apoptosis and decreased ingress of circulating monocytes into the joint are responsible for the initial reduction in the number of macrophages following infliximab treatment in hTNF-Tg mice [16]. Ly6C^hi^ monocytes exacerbate acute arthritic symptoms by transporting Murid herpesvirus 68 (MHV-68), a mouse virus closely related to the Epstein-Barr virus (EBV), into the inflamed joints of arthritic mice [17]. In contrast, NR4A1-dependent Ly6C^lo^ monocytes ameliorate joint inflammation in arthritic mice through the action of Treg cells [18]. However, Buckley et al. demonstrated that Ly6C^−^ monocytes also drive the development of inflammatory arthritis in mice [19]. These studies suggested that Ly6C^hi^, Ly6C^lo^, and Ly6C^−^ monocytes play different roles in the developmental process of arthritis. Therefore, these subsets of monocytes should be compared further in a mice model.

F4/80 is another marker for murine macrophages [20]. Mice with *Flip* deleted in myeloid cells expressing Lysozyme M (Flip^f/f^LysM^c/+^) developed more severe arthritis early in the clinical course; however, in such mice, peak arthritis was attenuated and the resolution phase was more complete. Prior to the induction of serum transfer-induced arthritis (STIA), murine SM numbers markedly decreased. At day 9 postarthritis induction, the number of F4/80^hi^ SMs in the joints of the Flip^f/f^LysM^c/+^ mice was increased. Flip was reduced in the F4/80^hi^ SMs in the ankles of the Flip^f/f^LysM^c/+^ mice, while the F4/80^hi^ population expressed an anti-inflammatory phenotype in both the Flip^f/f^LysM^c/+^ and control mice [21], suggesting that resident F4/80^+^ SMs show anti-inflammatory activities. However, we cannot exclude the effects from other myeloid cells because lysozyme M is also expressed in neutrophils and monocytes.

Based on these findings using SM markers, researchers attempted to alleviate RA severity by targeting SMs. Human umbilical cord blood stem cell-derived macrophages that are CD14^+^ (hUCB-derived MOs) can polarize and block inflammasome activation, alleviating RA [22]. Monoclonal antibodies against macrophage colony-stimulating factor (M-CSF) diminish the number of circulating intermediate and nonclassical (CD14^high^CD16^mid^/CD14^mid^CD16^high^) monocytes in RA patients [23]. SM (CD14^+^ and CD68^+^) depletion by clodronate-containing liposome injections can decrease expression of adhesion molecules (VCAM-1 and ICAM-1) in the lining layer of RA patients [24]. However, these methods are all designed to target total SMs without discriminating the different subsets of SMs. As suggested in a previous study [21], F4/80^+^ resident murine SMs show anti-inflammatory phenotype. Methods that eliminate total SMs could further amplify inflammation by diminishing the number of resident anti-inflammatory SMs in mice. Therefore, it would be more beneficial to specifically target the proinflammatory subset of SMs instead of total SMs.

In a rat RA model, multiple intraarticular injections of a custom Bordetella pertussis antigen and methylated bovine serum albumin (mBSA) in complete Freund's adjuvant boosted levels of both circulating SMs (ED1^+^) and resident SMs (ED2^+^) [25]. Richards et al. demonstrated [26] the increased efficiency of small unilamellar vesicles (SUVc) in reducing inflammation and joint destruction that was associated with a significant depletion of SMs from the rat synovial membrane. However, ED1, ED2, and ED3 were considered as markers for resident SMs in this study [26], which was not consistent with a previous study [25]. This inconsistency suggested that there is also no common marker for resident SMs in rat. Clodronate-laden liposomes induce long-term amelioration of RA rats, even if administered for a brief period during the florid phase of the disease. The amelioration is paralleled by the elimination of macrophages in immunocompetent areas of the spleen and draining lymph nodes, but not locally in the SMs (ED1^+^ED3^+^ resident SMs in rat synovium were unaffected). This study suggested that the treatment influenced the immunoregulation of rat SMs [27].

## 2. Current Knowledge

Currently, SM-based strategies only target total SMs, but not specific SM subsets. Given the potentially different functions of different SMs, it would be better to evaluate only one subset of SMs (boosting resident anti-inflammatory SMs or eliminating circulating proinflammatory SMs). All these results led to the following questions: how can different subsets of SMs be specifically identified and what is the functional difference between SMs from different origins in RA?

The historical review of macrophage ontology has been mainly obtained from circulating monocytes. However, many recently published studies have shown that macrophages have different origins at different tissues, including the embryonic yolk sac, fetal liver, and postnatal bone marrow [28–34]. As mentioned above, the different expressions of macrophage markers (CD14, CD68, CD163, MRP8, and MRP14) within the synovial lining and sublining layers also indicate the different origins of SMs. Although a few researchers have attempted to identify the role of a subset of macrophages in RA, no systematic analysis has been performed to identify SM ontology in either physiological or pathological conditions to date.

Apart from the established human and mouse macrophage markers mentioned above, some studies have identified several novel markers of SMs. The Z39Ig protein (complement receptor for C3b and iC3b) is expressed on resident tissue macrophages in various tissues, such as lung and liver [35, 36]. Z39Ig^+^cells appeared to be useful for identifying resident human SMs in normal synovium and the corresponding SMs in the synovial lining layer of inflammatory arthritis. Expansion of Z39Ig^+^CD11c^+^ cells is a characteristic of RA synovial lining layer [37]. The increased serum levels of leukocyte-derived granular proteins, lysozyme, and myeloperoxidase (MPO) in RA patients indicate a stimulated secretory activity of mononuclear phagocytes, including monocyte-derived macrophages [38]. Secreted stabilin-1 interacting chitinase-like protein (SI-CLP) functions as a regulator of the inflammatory response by BM-derived human macrophages [39]. In addition, prolactin receptor is expressed in RA and psoriatic arthritic synovial tissue and contributes to human SM (CD68^+^) activation [40]. Folate receptor beta acts as a human macrophage- (CD11b, CD14, CD16, and CD68-) mediated imaging marker and therapeutic target in RA [41]. Translocator protein acts as an imaging marker of human macrophage (CD163 and CD68) and stromal activation in RA pannus [42]. Macrophage mannose receptor (MMR) is highly expressed on BM-derived human macrophages. In synovial fluid of arthritic joints, MMR is expressed on CD11b^+^F4/80^+^ mouse macrophages [43]. However, these markers still need to be validated in animal RA models and a large cohort of RA patients. Collectively, the results from these related studies imply that SMs have at least two origins, namely, tissue resident and circulating monocyte-derived SMs, which significantly infiltrate the RA hyperplastic synovium. Therefore, we hypothesized that SMs demonstrate a mixed cellular population and different subsets of SMs may play different roles in RA.

Previous studies have indicated that the macrophage markers, F4/80 and CD11b, can be used to distinguish embryonic-resident and bone marrow-derived macrophages [28–33]. Thus, we attempted to identify embryonic-resident and bone marrow-derived SMs in the synovium [44]. Resident and bone marrow SMs were identified and showed distinct cellular features, including *in situ* proliferation, phagocytosis, and expression of proinflammatory and anti-inflammatory genes. In line with our expectations and previous reports, resident SMs exhibit anti-inflammatory and bone marrow SMs exhibit proinflammatory functions. However, more essential questions were raised from this study. What is the contribution of resident SMs from the yolk sac and fetal liver? What is the specific role of resident and bone marrow SMs in different developmental stages of RA? Therefore, the primary goal currently is to identify specific regulatory factors of resident and bone marrow SMs.

**Figure 1:**
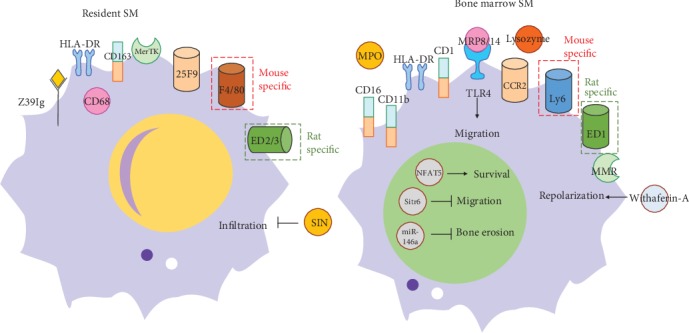
The markers comparison of resident SMs and bone marrow-derived SMs. (a) Resident SM markers include Z39Ig, CD68, CD163, MerTK, 25F9, F4/80, and ED2/3 (rat); (b) Bone marrow-derived SM markers include CD16, CD11b, CD14, MRP8/14, CCR2, Ly6C, MMR, MPO, lysozyme, and ED1 (rat). Up to now, these are several regulatory agents of resident SMs (SIN) and bone marrow-derived SMs. (NFAT5, Sirt6, miR-146a, MRP8/14, and Withaferin-A).

There are several proven regulatory factors of circulating SMs in arthritis. A key transcription factor, transcription factor nuclear factor of activated T-cells 5 (NFAT5), promotes macrophage (CD14^+^) survival in RA by inducing CCL2 secretion [45]. Myeloid sirtuin-6 (Sirt6) deficiency accelerates experimental RA by enhancing macrophage (LysM-Cre CD14^+^CD68^+^) activation and infiltration into the synovium. Mechanistically, Sirt6 deficiency in macrophages leads to inflammation with increases in acetylation and protein stability of forkhead box protein O1 (FoxO1) [46]. miR-146a serves as a key regulator of the differentiation of Ly6C^hi^, but not Ly6C^lo^, monocytes into osteoclasts under arthritic conditions by targeting the noncanonical NF-*κ*B family member RelB. The delivery of miR-146a to Ly6C^hi^ monocytes inhibits pathogenic bone erosion in CIA mice [47]. Increased macrophage (CD14^+^ from RA SF) activation is mediated through Toll-like receptors (TLRs) in RA [48]. Sinomenine (SIN), an active monomer obtained from the traditional Chinese medicine, Qingteng, attenuates proinflammatory SMs in the synovial tissue and ameliorates arthritis in mouse model [49]. In addition, Withaferin-A, a steroidal lactone encapsulated mannose decorated with liposomes, ameliorates RA by inducing SM (CD11b^+^) repolarization in adjuvant-induced arthritic rats [50]. Another interesting study showed direct evidence that SM (CD68^+^) depletion with clodronate-containing liposomes repressed the incidence and development of an antigen-induced arthritis model [51]. However, these studies only investigated the role of SMs as a homogeneous cellular group. Therefore, it would be considerably better if we can identify and evaluate these regulatory factors by comparing tissue-resident and monocyte-derived SMs, simultaneously (Figure 1).

## 3. Future Direction

SMs were previously named as macrophage-like synoviocytes (MLS, type A synoviocytes). It is thought that MLS and fibroblast-like synoviocytes (FLS, type B synoviocytes) are the two main cellular components in the synovium. However, as the understanding of SMs deepens, researchers have begun to notice that SMs are a specific group of macrophages within the synovium. The number of SMs significantly increases within inflammatory and hyperplastic synovium and they play an essential role in the pathogenesis of RA. Previous studies suggested that there are two subsets of SMs, namely, resident and infiltrating circulating SMs. The origins of the two SM subsets have been identified from the embryonic to postnatal stage. The different cellular properties and dynamic expression patterns of SMs in the synovium from RA patients/CIA mice imply that resident and bone marrow SMs play different roles in the development of arthritis [52].

**Figure 2:**
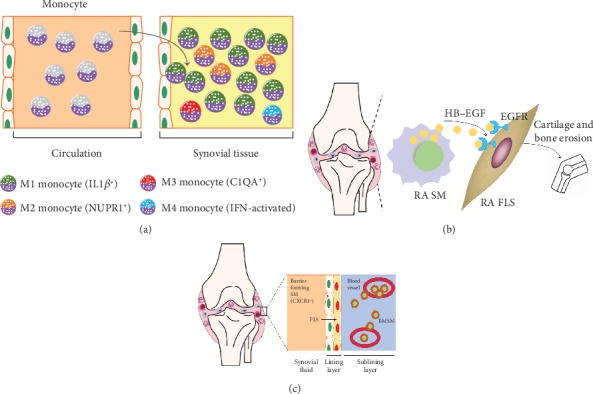
The heterogeneity of SMs in RA was discovered by single cell sequencing. (a) Accelerating Medicines Partnership Rheumatoid (AMPR) Arthritis consortium identified the unique activation states of synovial monocytes as four subsets: IL1*β*^+^ proinflammatory monocytes (M1), NUPR1^+^ monocytes (M2) with a mixture of leukocyte-poor RA cells, C1QA^+^ (M3), and IFN-activated monocytes (M4) by scRNA-seq analysis. (b) HBEGF^+^ inflammatory SMs are enriched in RA tissues and are shaped by FLS. These SMs promoted fibroblast invasiveness in an EGFR-dependent manner, indicating that intercellular cross talk in this inflamed setting reshapes both cell types and contributes to FLS-mediated cartilage and bone erosion. (c) Culemann *et al*. found that certain SMs form a cell layer that protects joints from the inflammatory immune-cell attacks on bone and cartilage. This barrier is formed in the lining layer (next to FLS). The barrier-forming SMs express proteins associated with a type of barrier-forming epithelial cell, and these proteins form structures called tight junctions. Barrier-forming SMs arise from a type of macrophage called an interstitial macrophage, which resides in the sublining layer. By contrast, nonresident macrophages enter the joint from blood vessels. These cells, which can drive inflammation, arise from monocytes.

Recently, numerous studies have been published that illustrated the heterogeneity of SMs in RA using single cell sequencing (Figure 2). Among these studies, high-dimensional single-cell datasets from synovial tissues of RA patients have been published by the Accelerating Medicines Partnership Rheumatoid (AMPR) Arthritis consortium [53]. Kuo et al. identified that HBDGF^+^ inflammatory SMs could induce FLS invasiveness in synovial tissues from RA patients [54]. In addition, murine CX3CR1^+^ barrier-forming SMs have also been identified [55]. Interestingly, the cellular characteristics of HBDGF^+^ inflammatory and CX3CR1^+^-renewing SMs are markedly similar to bone marrow and resident SMs, respectively. Accordingly, it seems that the heterogeneity of SMs might be considerably more complicated than previously thought. In the future, we will further interpret the subsets of resident SMs, such as from the yolk sac and fetal liver, and the specific functions of different subsets of SMs in RA using single cell sequencing, which will lay the foundation of RA therapy based on targeting different SM subsets.

Although some macrophage-targeted treatments have shown ameliorating effects on animal arthritis models, such as depleting macrophages with clodronate-containing liposome, none of them have reached the stage of clinical trials. One potential obstacle may be that these macrophage-targeted therapies do not specifically target different subsets of macrophages, i.e., both proinflammatory and anti-inflammatory macrophages are eliminated simultaneously. A future direction for RA therapy would be to develop therapies based on the different SM subsets. Most importantly, specific markers or targets of SMs with different origins need to be identified. To this end, several “omics” studies at different molecular levels, including transcriptome, epigenome, proteomics, and metabiomics, should be performed to compare different SMs. For example, in order to further determine the origin, subsets and dynamics of SMs, advanced technology, such as combing and fate-mapping/parabiosis mice models and 10 × genomic single-cell RNA sequencing, should be performed to identify the dynamic molecular characteristics of individual SMs in the joint synovium. The significant and dynamic changes between subsets of SM could be detected by applying this technique, even for seemingly homogeneous resident SMs or circulating-derived SM populations. Firstly, this technology will provide a lot of undiscovered information about different roles of SMs from different origins in RA. Secondly, proteomic measurements also should be tested at the same time as an important validation. In particular, protease measurements of individual SMs are important for optimal understanding of cellular signaling encoded in posttranslational protein modifications. In addition, single-cell technique is ideal for clinical sample analysis because it requires only a small number of biomaterials.

There are at least two SM-based treatments of RA based on the cellular features of resident and bone marrow-derived SMs. The first one is aimed at preventing the reduction of anti-inflammatory resident SMs, whereas the other one is aimed at impeding the hyperinfiltration of proinflammatory bone marrow-derived SMs in the RA joint synovium. Macrophage-based therapies in RA have not provided any promising results to date; however, the identification of different origins and functions may provide novel ideas and directions for this field. A series of previous high-quality articles demonstrated that resident SMs and bone marrow-derived SMs show significant cellular differences in RA. Further investigation is required to illustrate the specific characteristics of these two SMs, develop different RA treatment strategies from the perspective of their anti-inflammatory and anti-inflammatory properties, and look for novel targets with high specificity and low side effects.

## Figures and Tables

**Table 1:** The functions of SMs in RA synovium.

Cell type	Mediator	Function
Polarization		
SMs (M1)	TF and pathway: STAT1, IRF5, SOCS1, NF-*κ*B pathway	Proinflammation; glycolysis; iron retention
	Cytokine: IL-1*β*, TNF-*α*, IL-12, IL-23	
	Chemokine: CXCL9/10/11, CCL5	
	Surface marker: MHCII	
SMs (M2)	TF and pathway: STAT6, IRF4, SOCS3, KLF4, c-Myc	Anti-inflammation; oxidative phosphorylation; iron export
	Cytokine: IL-4, IL-10	
	Chemokine: CCL17/22	
	Surface marker:CD206, CD163,MGL	
Cell-cell communications		
Synovial fibroblasts (SF)	IL-1*β*, TNF-*α*	SMs promote SF proliferation.
Osteoclasts	IL-1*β*, IL-6, TNF-*α*	SMs promote osteoclasts activation.
Monocytes	IL-1*β*, IL-8, TNF-*α*, CCL2	SMs recruit monocytes.
Neutrophils	IL-1*β*, IL-8, TNF-*α*, CCL2	SMs recruit neutrophils.
T cells (Th1 cells)	TNF-*α*, IL-12	SMs promote Th1 polarization.
T cells (Th17 cells)	IL-23	SMs promote Th17 polarization.
B cells	Immune complex and autoantibody	B cells activate SMs.
